# Integration of Clinical and Gene Expression Data Has a Synergetic Effect on Predicting Breast Cancer Outcome

**DOI:** 10.1371/journal.pone.0040358

**Published:** 2012-07-11

**Authors:** Martin H. van Vliet, Hugo M. Horlings, Marc J. van de Vijver, Marcel J. T. Reinders, Lodewyk F. A. Wessels

**Affiliations:** 1 Delft Bioinformatics Laboratory, Faculty of Electrical Engineering, Mathematics and Computer Science, Delft University of Technology, Mekelweg, Delft, The Netherlands; 2 Department of Pathology, Academic Medical Center, Meibergdreef, Amsterdam, The Netherlands; 3 Bioinformatics and Statistics group, Department of Molecular Biology, Netherlands Cancer Institute, Plesmanlaan, Amsterdam, The Netherlands; Duke-National University of Singapore Graduate Medical School, Singapore

## Abstract

Breast cancer outcome can be predicted using models derived from gene expression data or clinical data. Only a few studies have created a single prediction model using both gene expression and clinical data. These studies often remain inconclusive regarding an obtained improvement in prediction performance. We rigorously compare three different integration strategies (early, intermediate, and late integration) as well as classifiers employing no integration (only one data type) using five classifiers of varying complexity. We perform our analysis on a set of 295 breast cancer samples, for which gene expression data and an extensive set of clinical parameters are available as well as four breast cancer datasets containing 521 samples that we used as independent validation.mOn the 295 samples, a nearest mean classifier employing a logical OR operation (late integration) on clinical and expression classifiers significantly outperforms all other classifiers. Moreover, regardless of the integration strategy, the nearest mean classifier achieves the best performance. All five classifiers achieve their best performance when integrating clinical and expression data. Repeating the experiments using the 521 samples from the four independent validation datasets also indicated a significant performance improvement when integrating clinical and gene expression data. Whether integration also improves performances on other datasets (e.g. other tumor types) has not been investigated, but seems worthwhile pursuing. Our work suggests that future models for predicting breast cancer outcome should exploit both data types by employing a late OR or intermediate integration strategy based on nearest mean classifiers.

## Introduction

Many predictors of breast cancer outcome have been published. These predictors have been derived from gene expression data, such as the 70-gene (Veer *et al.* [1]), and 76-gene (Wang *et al.* [2]) signatures, or clinical data, such as the Nottingham Prognostic Index (NPI, [3]) and AdjuvantOnline! tools [4]. A few studies have aimed at training a model using both of these data types. In doing so, several approaches were followed, that we outline below.

First of all, the clinical data can be used as a means to stratify patients in subgroups, and then train a gene expression predictor in each of the subgroups. For instance, Wang *et al.* [2] and Teschendorff *et al.* [5] have trained a gene expression classifier for ER positive, and separately for ER negative patients [6]. Alternatively, multiple clinical parameters can be used as the initial stratification. For example, Dai *et al.* [7] stratified into ER/Age-high, and ER/Age-low. Stratifications for ER and HER2 have also been made using gene expression data rather than clinical data, which could lead to better prognostic value [8]. Most of these studies have employed a set of standard clinical variables, such as ER status, tumor grade, tumor size, etc. Horlings *et al.* (In preparation, [9]) have characterized additional clinical features (e.g. matrix formation, central fibrosis, etc.) for an existing cohort of 295 breast cancer samples [10]. By themselves, these additional clinical variables have independent prognostic power. However, if and how this power can be used to build a better classifier for outcome prediction has not been investigated.

Gevaert *et al.* [11] have used a Bayesian framework to combine expression and clinical data. They found that decision integration (combination of the outputs of Bayesian classifiers trained on either data type), and partial integration (structure learned per data type, parameters learned after combining the data types) lead to a better performance, whereas full integration (concatenation of the two data types, followed by training the model on the complete set) showed no improvement. These results were obtained by using a cross validation approach on the 78 samples in the Veer *et al.* [1] dataset. However, on the 19 sample validation set from the same study the pure gene expression based classifier (i.e. no integration) performs slightly better. A major concern in their analysis is that a supervised preselection of genes is performed on the entire dataset, resulting in a potential bias [12]. On the same dataset, Boulesteix *et al.* [13] employed a random forests and partial least squares approached to combine expression and clinical data. In contrast, Boulesteix *et al.* [13] reported that microarray data do not noticeably improve the prediction accuracy yielded by clinical parameters alone.

Daemen *et al.* [14] pursued an intermediate integration approach based on combining kernels (kernel inner product matrices derived from the separate data types) for application in a Support Vector Machine (SVM). They applied their method to the 295 breast cancer sample dataset from Vijver *et al.* [10]. For performance assessment, they employed a train-test setup which was not repeated, i.e. no cross-validation was performed which has been shown to be necessary to obtain realistic performance estimates (Michiels *et al.* [15]). This setup was shown to outperform classical diagnostic systems (e.g. StGallen, National Institute of Health (NIH) and Nottingham Prognostic Index (NPI)), but shows comparable performance to single data type models.

Pittman *et al.* [16] combined clinical and expression data for predicting breast cancer outcome by means of a tree classifier. This tree classifier was trained using meta-genes and/or clinical data as inputs. A proper cross validation was performed, but no clear indication of a performance improvement is given.

All of the existing studies together are inconclusive as to whether the combination of expression and clinical data leads to better classifiers for predicting breast cancer outcome. Therefore, we perform a rigorous evaluation using five classifiers of varying complexity, three different integration strategies, and compare them to models trained on each data type separately. We assess the performance using a double loop cross validation protocol allowing an unbiased comparison. We use a breast cancer dataset, for which we have expression data [10], and an extensive collection of clinical data (Horlings *et al.*, In preparation, [9]). Moreover, we use four independent breast cancer datasets for validation of the obtained classifiers [17]. We show that all classifiers perform better when used in conjunction with an integration strategy. More specifically, the late OR integration strategy is the overall best strategy. Interestingly, classifiers trained on each data type separately have an almost equal performance.

## Materials and Methods

### Vijver Dataset

We have used the 295 breast cancer sample dataset from Vijver *et al.* [10]. For all 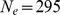 samples microarray data is available. We selected the 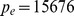 probes with an Entrez identifier. From this dataset, we selected 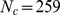 samples, which we could assign to a poor/good outcome group based on their survival characteristics (poor: event within five years of follow up, good: at least five years of metastasis free survival), a dichotomization commonly made, e.g. Veer *et al.* [1]. Thus, the remaining 36 samples were not included in the dataset since these have been censored before five years of follow up, making it impossible to assign them to the correct outcome group. Throughout this paper, we will refer to the expression data as ‘E’.

In addition to expression data, we have a variety of clinical data available (Horlings *et al.*, In preparation, [9]). The clinical features include the originally published variables (e.g. Grade, Age, ER status, etc), outputs from clinical models (e.g. NPI, StGallen, and Adjuvant), complemented with a set of novel pathological variables (e.g. Matrix Formation, Central Fibrosis, etc.). Table S1 shows a complete list and details of the clinical variables used. In total, we considered 45 clinical variables (which have no missing values for these 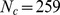 samples), of which 2 were nominal, 33 were binary or ordinal, and 10 were continuous. The two nominal variables were converted into binary features, i.e. one feature per group in the original nominal clinical variable. This way, we obtained a total of 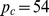 clinical features. Throughout this paper, we’ll refer to the clinical data as ‘C’.

We applied mean-variance normalization per feature, per dataset (i.e. for both E and C) to ensure approximately equal spread for all features.

### Other Datasets

Reyal *et al.* [17] have compiled a collection of six datasets, leading to a total of 947 breast cancer samples. From this compendium we have extracted the samples for which Age, Tumor Size, Grade, ER status, Lymph Node status as well as the poor/good survival label (using the same 5 year threshold as for the Vijver datset) were available. This lead to a total of 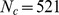 samples (107 poor, 414 good) from the Desmedt *et al.* [18], Miller *et al.* [19], Loi *et al.* [20], and Chin *et al.* [21] datasets. The NPI was calculated using these clinical parameters as previously defined [22], and both the continuous as well as discretized NPI were appended to the clinical data. Thus, a total of seven clinical parameters, 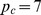 (this is much less than the 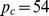 in the Vijver dataset), were available for all 521 samples. For the expression data we used the probes that were also present in the Vijver dataset, by matching Entrez ids (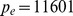). After this selection, we applied mean-variance normalization per feature, per dataset (i.e. for both E and C).

### Classifiers

We employed five classifiers with varying degrees of complexity, some of which have been used before to integrate clinical and expression data. We shortly discuss each classifier (see Table 1):

**Table 1:** Overview of the combinations of classifiers and integration strategies that were tested.

	Type of integration			
Classifier	None	Early	Intermediate	Late
NMC	+	+	+	+
SBC	+	+	+	+
3NN	+	+	+	+
SVM	+	+	+	+
Tree1 (No feature selection)	+	+	–	+
Tree2 (No pruning)	+	+	–	+
HybridTree (C)	–	–	+	–
HybridTree (E)	–	–	+	–

Tested combinations are indicated with a ‘+’, those not tested with a ‘–’. The methods with no integration were applied to both the expression and clinical data separately.

1. **A Nearest Mean Classifier (NMC), with the cosine correlation as distance measure.** This linear classifier has previously been applied on expression data, and was shown to outperform more complex classifiers [1], [23].
2. **A Simple Bayes Classifier (SBC)** [**24**] **, which is based on the assumption that the features are independent.** This simplifies the computation of the class conditional densities significantly. In spite of this simplification, it has been shown that this classifier performs remarkably well [24]. Class continuous densities of continuous features were modeled using Gaussian distributions.
3. **A 3-Nearest-Neighbor classifier (3NN)** [**25**] **, employing the cosine correlation as distance measure, and majority voting to assign a sample to a class.** Since there is a class imbalance, the majority vote is adjusted with the class priors. This classifier is capable of constructing non-linear decision boundaries. Moreover, it is frequently applied to microarray data, see e.g. Dudoit *et al.* [26].
4. **A Support Vector Machine (SVM)** [**27**] **, using a cosine correlation kernel** [**14**] **, i.e. a kernel function which computes the cosine correlation between two input objects.** This classifier is appropriate for small sample size problems, and has previously been used to integrate expression and clinical data [14]. The cosine correlation kernel for SVMs is identical to a linear kernel, where the feature vector for each sample has been divided by its L2-norm [28]. The C parameter was fixed at 1 (default value, svmtrain in Matlab R2012a). To account for class imbalance, C was rescaled by 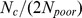 for the samples in the poor group and by 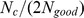 for the samples in the good group.
5. **A Tree classifier (Tree)** [**29**] **, which allows for highly non-linear decision boundaries.** Gini’s diversity index was used as splitting criterion. In order to regularize the tree classifier, we employed two variants. The first variant (Tree1), optimizes the tree depth but selects a subset of features from all features. The second variant (Tree2) is not pruned, but selects features from the subset of up to 200 most predictive features as provided by the feature selection procedure.

We excluded the Bayesian approach introduced by Gevaert *et* *al.* [11], since it is computationally intractable to train this model on all genes.

### Cross Validation Setup

To evaluate the performance of the classifiers, and determine the optimal number of features (tree depth for the Tree and the HybridTree classifiers (see Section ‘Integration strategies’)), we applied a double loop cross validation protocol (DLCV, Wessels *et* *al.* [23]). The DLCV procedure employs two loops, an outer loop for validation purposes to estimate the performance on a left, out independent part of the data, and an inner loop in which the classifier’s parameters are optimized. The DLCV procedure can be described in a few steps:

1. **For each repeat, the data is split (stratified) into five parts (different splits for each repeat).**
2. **For each fold, four parts are used for the inner loop (training set), the fifth part is used in the outer loop for validation (validation set).**
3. **On the training set, a 10-fold cross validation is performed to estimate the optimal number of features (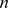 is defined as the number of genes at which the 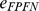 is minimal) to be used in the classifier, i.e.** the number of features that resulted in the best classification performance based on the 10-fold cross validation.
4. **Next, a classifier is trained on the complete training set, using the estimated optimal number of features.**
5. **Finally, the performance of that classifier is assessed on the validation set.**

Typically, datasets are imbalanced in the sense that the samples from the classes do not appear in equal fractions in the dataset. Moreover, the imbalance will be different for different datasets. Hence, directly comparing overall error rates (fraction of wrong assignments), is not an appropriate comparative measure. Therefore, classification errors were calculated by using the average False Positive False Negative ratio, defined as:
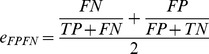
(1)where 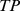 represents the number of true positives, 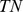 the number of true negatives, 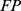 the number of false positives, and 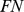 the number of false negatives. This ratio is equivalent to 1-.5 (Sensitivity + Specificity).

The entire protocol was run 60 times (i.e. 60 repeats of the double loop cross-validation protocol). To find the optimal number of features, we constructed learning curves in the inner loop for up to 200 features (or 54 when only using the clinical data). In all experiments, we used the exact same repeats and folds. As a result, we were able to compare the performance results in the outer loop on a pair-wise basis, using a one-sided, paired t-test.

Kaplan-Meier curves were constructed by using the predictions that were made in the outer loop. Consequently, in each repeat, every sample has once been part of the test set in the outer loop. Thus, for each sample we have a fully unbiased prediction of the binary label. After completing the 60 repeats, we have 60 unbiased predictions of each sample. Next, we take the mean of those 60 predictions, and assign a sample to the poor group if the average is below 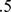 and to the good group when the average is above 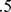. This approach is known as the ‘pre-validation strategy’ [30]. The predictions are independent, but nevertheless the training sets will overlap in terms of samples. However, this only yields a small bias [30].

As an alternative performance criterion, we also considered the AUC (Area Under the Curve) of the ROC (Receiver Operator Characteristic) curve instead of 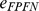. We employed the perfcurve function in Matlab, which tests all possible thresholds on the vector of classifier output scores, and then uses trapezoidal approximation to estimate the area under the curve (AUC). The ROC analysis can straightforwardly be applied to the ‘early’, ‘intermediate’, and ‘no integration’ setups. Using the vector of scores obtained from the classifier, we varied the threshold in steps of 1 sample. However, the late integration setups require two binary vectors, and thus require choosing an operating point on each of the two separate classifiers. This complicates the construction of an ROC curve. We solved this problem as follows. Each classifier outputs a ranking of the samples from most likely to least likely poor outcome. For N samples this results in a total of 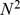 possible thresholds (ROC operating points) for the joint classifier. Rather than considering all these possibilities, we only considered operating points where both classifiers assign the same number of samples to the poor (and good) outcome class, resulting in 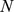 joint operating points. So, for the 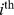 operating point, we set the threshold on both the the E and C classifier such that 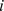 samples are classified as poor outcome. This results in two binary vectors, both with 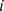 values set to 1 (poor outcome) and the rest to 0 (good outcome). After that the two vectors of binary prediction labels are combined using the AND/OR operator, and compared against the true label to provide the sensitivity/specificity coordinates for the ROC curve.

### Feature Selection

In the inner loop of the cross validation procedure, we used a feature filtering approach. To rank the features, we employed a t-test for the continuous features and the chi-squared test for discrete features. The combined set of features are then ranked based on the p-values of the associated tests.

### Integration Strategies

**Figure 1:**
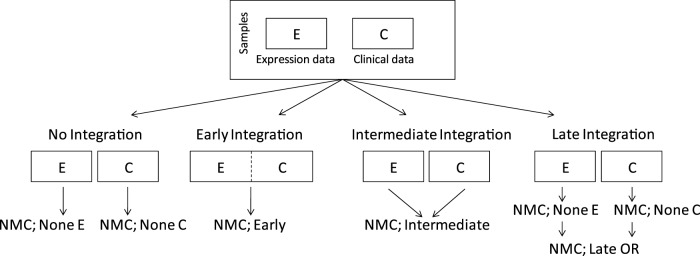
Schematic indication of the expression dataset (E), clinical dataset (C), along with different integration strategies that were tested. Examples are shown for the NMC classifier. On the left, we depict the ‘no integration’ setup, for which a separate classifier is trained on each dataset (‘NMC; None E’ and ‘NMC; None C’). For early integration, the two datasets are concatenated into EC, on which a single classifier is trained (‘NMC; Early’). Similarly, for intermediate integration, the datasets are combined at an intermediate step in learning the classifier (‘NMC; Intermediate’). Finally, late integration is depicted on the right, where a classifier is trained on each dataset separately, and combined by means of a logical function (‘NMC; Late OR’).

Following Gevaert *et al.* [11], we considered early, intermediate and late integration. In Figure 1 we depict each of the three strategies, and describe them below. Table 1 shows which integration strategies are considered in combination with which classifiers.

Classifiers are indicated by their abbreviation, followed by the type of integration used. For example, ‘NMC; None E’ is the nearest mean classifier (NMC) without integration (None), trained on expression data (E).

### Early Integration

For the early integration strategy we concatenated the E and C datasets, and thereby created a single dataset, EC, with 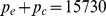 features. Classifiers trained on EC are indicated with the suffix ‘Early’, e.g. ‘NMC; Early for the NMC variant.

**Figure 2:**
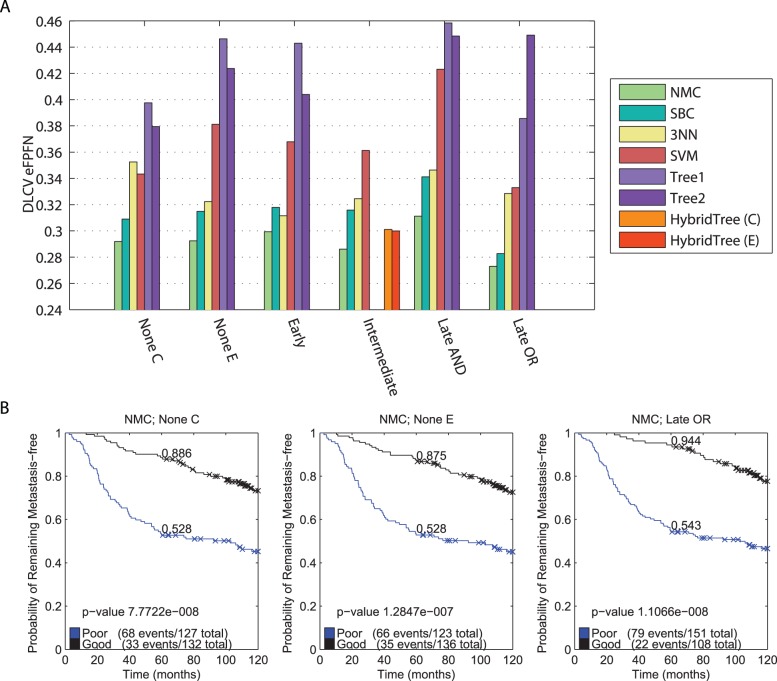
Error rate of the different classifiers and integration strategies. A) Bar plot indicating the average DLCV eFPFN errors obtained using the different classifiers, integration strategies, and types of input data. B) Kaplan-Meier curves of the NMC classifier without integration, and the one using the Late OR integration strategy. We’ve indicated the p-value from the logrank test, and the fraction at five years.

**Figure 3:**
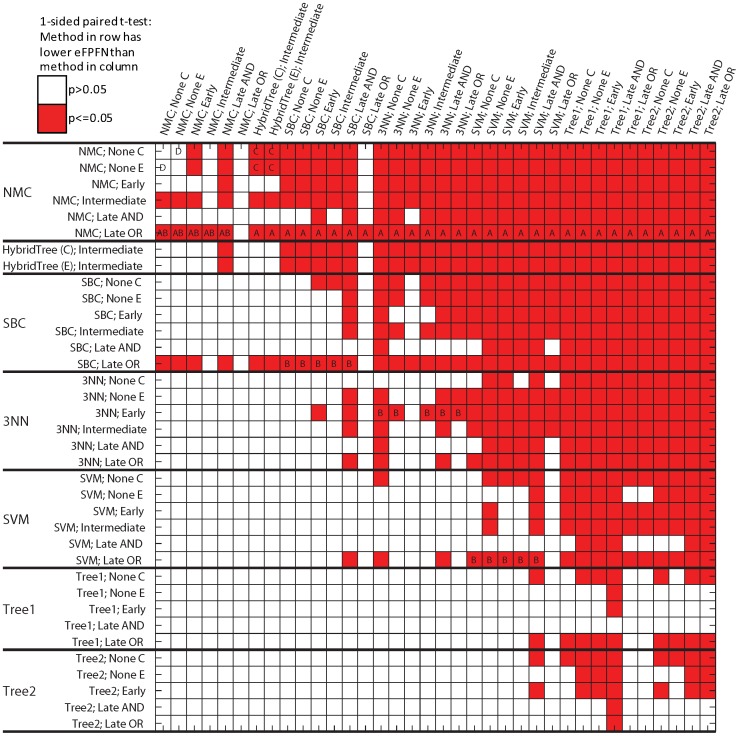
Overview of all pairwise comparisons of the classifiers. Comparisons were made by means of a one sided, paired t-test, testing the hypothesis that the error associated with the approach listed in the row is lower than the error associated with the approach listed in the column. Red cell shading indicates a p-value smaller than 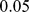, and white cell shading indicates that the p-value was larger than 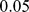. Letters in the cell refer to particular comparisons that are discussed in the text.

### Intermediate Integration

For all classifiers (except the intermediate Tree classifier), we first determine the optimal sets of features from each data type separately (in the inner loop). In all subsequent steps, we used these sets of features and all training samples. We define 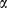 as a mixing parameter (ranging from 0 to 1) and 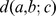 as the cosine correlation between vectors 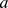 and 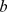 using the optimal features in 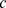. What we do for each classifier is described below:

1. **For the NMC classifier, we compute the centroids of the poor and good class (denoted as 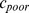 and 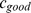), for both the E and C data types separately.** Next, for a sample 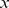, we compute a combined distance (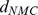) to the centroids, which is a linear function of the distances in the individual spaces, and is formulated as:

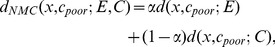
(2)

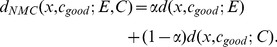
(3)


Subsequently, the sample 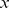 is assigned to the class for which the distance 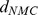 is the smallest.

1. **For the SBC classifier, we first computed the posterior probabilities of the poor and good class, for both data types, given a sample 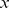.** The result is denoted as 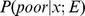, 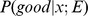, 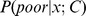, and 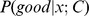, where 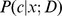 denotes the probability that sample 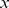 is in class 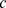 given the data in 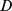). Next, the overall posterior probability (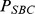) is computed as a linear combination of the individual posteriors:

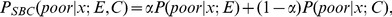
(4)

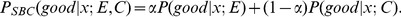
(5)


Subsequently, the sample 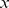 is assigned to the class with maximal posterior probability.

1. **For the 3NN classifier, we first calculated the distance of a sample 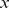 to a training sample 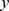 in E and C, leading to 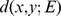, and 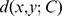.** Next, the overall distance 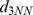 is computed as a linear combination of the individual distances:

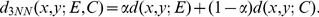
(6)After calculating the distance, 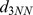, from 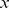 to all training samples 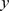, 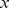 is assigned to the class most frequently occurring amongst the three closest samples (the majority vote is adjusted with the class priors).

1. **For the SVM classifier, we use the cosine correlation to compute a kernel 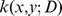, the kernel distance between samples 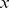 and 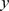 given data type 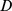.** We then construct a new kernel matrix 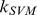 by taking a linear combination of the kernel matrices from the separate data types:

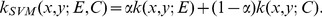
(7)For computational tractability, positive semi-definiteness of the kernel has to be ensured (Mercer conditions). This is the case when the weights employed in the linear combination (7) are non-negative [14], a condition which is satisfied here (cosine correlation as kernel).

1. **For the Tree classifier, we followed an approach similar to Pittman** ***et al.*** **** [**16**] **.** First, we considered a method where we start with a NMC trained on C (since this is a computationally inexpensive classifier with known good performance). This classifier splits the samples into two groups, each associated with a node in the tree. In these and all subsequent nodes, we branch further using a NMC trained on E and the samples at the relevant node. The procedure was stopped when a particular branch was pure (only poor or only good samples), or contained fewer than ten samples. This approach will be referred to as HybridTree (C). We also included the complementary setup, which starts with a NMC trained on E, and uses NMC classifiers trained on C in the subsequent nodes (HybridTree (E)).

For the two HybridTree variants, we optimized the tree depth in the cross validation procedure (inner loop), while we fixed the number of features used in each classifier to the top 100 features when trained on E, and the top ten features when trained on C. These features were selected using all training samples (in the inner cross validation loop) in a particular branch of the tree, using the same feature selection methods as described above in the Section ‘Feature selection’.

**Figure 4:**
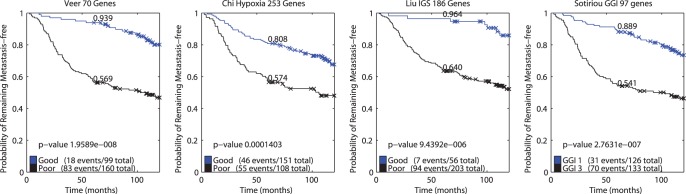
Kaplan-Meier curves of the same 259 sample subset from the Vijver dataset, employing four different signatures. P-values reflect the logrank test.

The mixing parameter 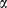 that several intermediate integration strategies use, is also optimized in the inner loop, now using the entire training set. More specifically, we vary 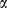 from 0 to 1 in steps of 0.01, and then inspect the error on the training data. This ensures that the 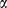 parameter is optimized in an unbiased fashion since the test samples in the outer loop are not involved in optimizing 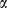.

**Figure 5:**
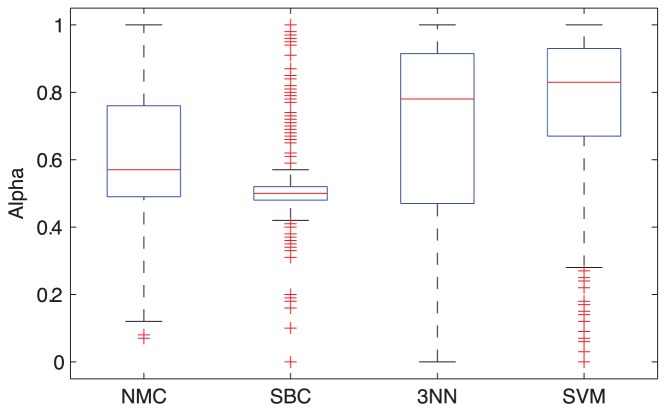
Boxplot showing the 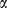 values that are obtained using the different classifiers with an intermediate integration strategy (300 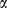 values from the 60 repeats of 5 folds).

Classifiers trained using the intermediate integration strategy are indicated with the suffix ‘Intermediate’, e.g. ‘NMC; Intermediate’ for the NMC variant.

### Late Integration

For late integration we train a classifier on E and C separately. After that, we apply a logical function on the binary classifier outputs (poor is positive, and good is negative). We considered a logical AND function, for example for the NMC classifier::
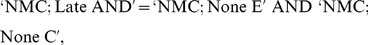
(8)and a logical OR function, for example for the NMC classifier::



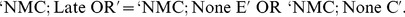
(9)The difference between the logical AND and OR functions is the way the discordantly classified samples are treated. Using the AND function these are assigned to the good class, and using the OR function these are assigned to the poor class. These two options are formally known as ‘believe the positive’ (OR) and ‘believe the negative’ (AND) integration [31].

**Figure 6:**
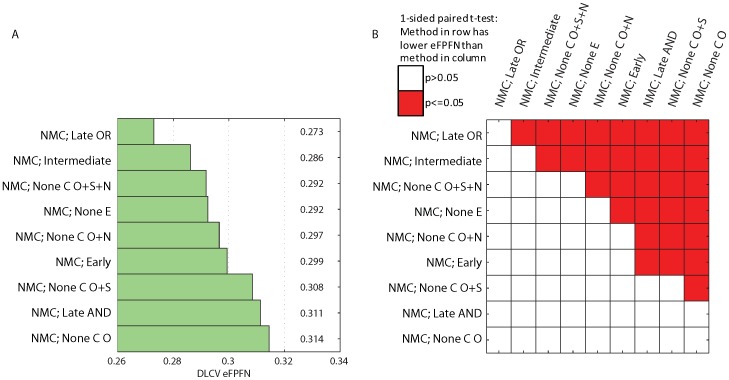
Error rate of the NMC classifier using different (subsets) of the E and C as input. A) Bar plot indicating the average DLCV eFPFN errors obtained using the NMC classifier with different integration strategies, and types of input data. B) Overview of all pairwise comparisons of the NMC classifiers, by means of a one sided, paired t-test, testing the hypothesis that the error associated with the approach listed in the row is lower than the error associated with the approach listed in the column. Red cell shading indicates a p-value smaller than 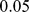, and white cell shading indicates that the p-value was larger than 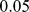.

## Results

### The ‘NMC; Late OR’ Classifier Performs the Best

The lowest error is achieved using the NMC classifier with late OR integration strategy (‘NMC; Late OR’, eFPFN = 0.273, Figure 2A. Figure 3 (squares indicated with an ‘A’) shows that this error is significantly lower than all other classifiers. This is a clear indication that there is synergy between the two data types, and that the late OR integration strategy provides a way to exploit the synergy. In addition, Figure 2B shows that the Kaplan-Meier curve of the ‘NMC; Late OR’ classifier is more significant than those from the NMC classifiers trained on a single data type. More specifically, the good group has become purer at the five year point (94.4% metastasis event free, versus 87.5% and 88.6%, respectively).

**Figure 7:**
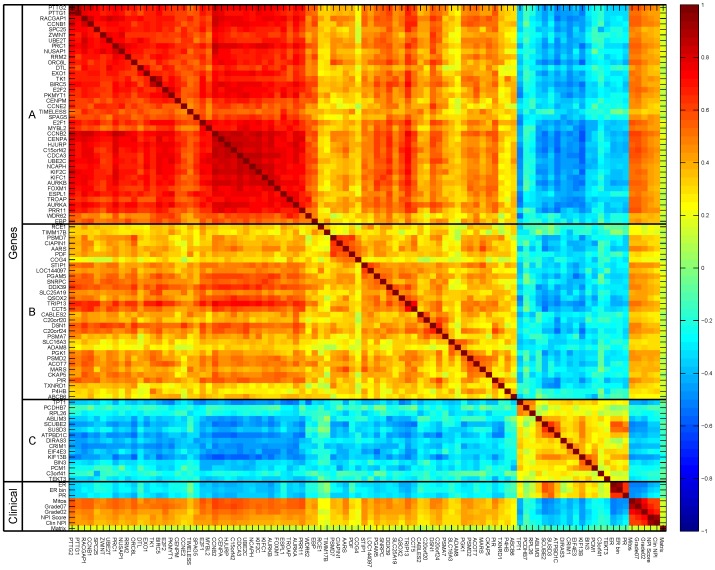
Heatmap showing the Pearson correlation of the 87 genes, and nine clinical features used by the ‘NMC; Late OR’ classifier. On the left, the genes and clinical parameters are indicated, along with three subgroups of genes (labeled A, B, and C), that form the main clusters of genes. The color in the heatmap scales with the correlation of a particular pair of features, and ranges from -1 (blue) to 1 (red).

Figure 4 shows the Kaplan-Meier curves of four other signatures that were applied to the same set of 259 samples from the Vijver dataset (70-gene signature, Veer *et al.* [1]; 253-gene hypoxia signature, Chi *et al.* [32]; 186-gene invasiveness signature, Liu *et al.* [33]; 97-gene genomic grade index signature, Sotiriou *et al.* [34]). The p-value of the ‘NMC; Late OR’ Kaplan-Meier curve is lower than each of these other four signatures. That is, the ‘NMC; Late OR’ strategy performs comparable to or better than all these signatures as measured by either the significance of the log-rank p-value or the fraction of patients that remain metastasis free at 10 years. This is especially noteworthy in the case of the 70 genes as this signature was trained on a subset of de Vijver dataset, and is therefore expected to be positively biased.

**Figure 8:**
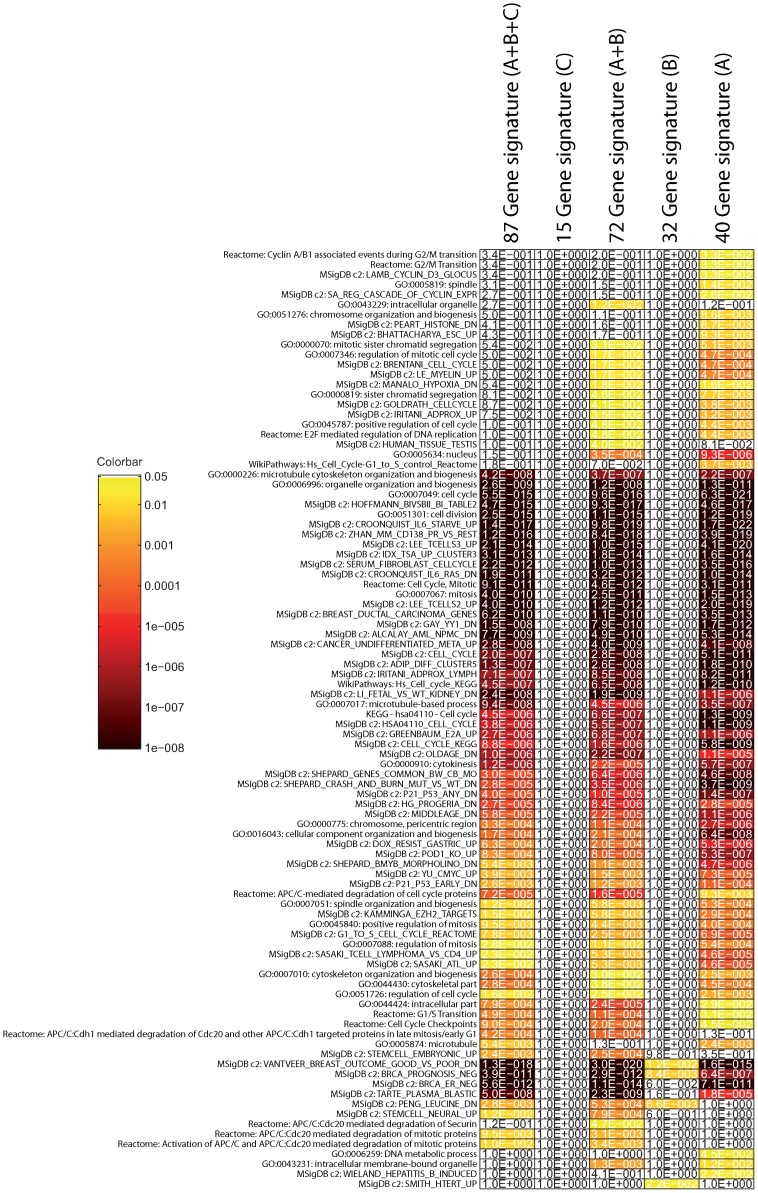
Enrichment of the 87-gene signature, and the three identified subgroups of genes A, B and C (groups defined in **Figure 7** based on clustering). Cell shading in the heatmap shows the Bonferroni corrected p-value of the enrichment (hypergeometric test), white corresponds to a p-value larger than 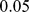 and the color ranges from just below 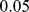 (yellow) to 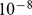 or lower (dark red)) on a logarithmic scale, as indicated in the colorbar.

### Integration Improves Performance

The NMC, SBC, SVM, and Tree1 classifiers perform the best when employing the late OR integration strategy, whereas the 3NN classifier performs the best when employing the early integration (see Figure 3, squares indicated with a ‘B’). In addition, the median mixing parameter 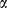 that was selected in the intermediate approaches is around .5 or higher (see Figure 5), suggesting that both data types are important. Thus, integration of the two data types proves beneficial for all classifiers with the ‘Late-OR’ strategy resulting in the best performance for all classifiers except the 3-NN classifier.

### Less Complex Classifiers Outperform Complex Classifiers

Figure 3 shows that the NMC classifier outperforms all other classifiers, with the exception of the ‘SBC; Late OR’ option. Overall, we can approximately rank the classifiers based on the achieved error rates in the following order: NMC 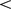 HybridTree 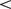 SBC 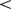 3NN 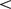 SVM (with the remark that the C parameter of the SVM has not been optimized) 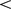 Tree. This ordering correlates with the complexity of the classifiers, and confirms previous results [23], [35]. The most likely explanation for this ordering is the small sample size problem, due to which the more complex classifiers run into overtraining problems, and consequently perform worse on independent data.

**Table 2:** List of the nine clinical variables selected in the ‘NMC; Late OR’ classifier.

Label	Description
Mitos	Subscore from grade
Grade07	Grade assessed in 2007
Matrix	Matrix formation
Grade02	Grade assessed in 2002
NPI Score	Continuous score from NPI
ER	Percentage of ER positive cells
ER bin	Discretized ER status (positive when above 10%)
Clin NPI	Discretized score from NPI
PR	Percentage of PR positive cells

### A Hybrid Tree Approach is not Useful on Breast Cancer Datasets

The average tree depth that is selected when using the HybridTree (C) classifier is 1.1. At this tree depth, the HybridTree (C) is practically equivalent to the NMC using clinical data. On the other hand, the HybridTree (E) has an optimal tree depth of 1.8. This suggests that a second level of NMCs using clinical features might be beneficial on top of the expression NMC. However, both HybridTree classifiers are significantly outperformed by the NMC classifiers without any integration (Figure 3, indicated with a ‘C’). We suspect that this is due to the extremely small numbers of samples available in the second layer and further down the tree. The classifiers in these nodes are most likely highly overtrained and consequently do not generalize very well.

The HybridTree (C) setup is very similar to training an expression based classifier within clinical subgroups. Our analysis indicates that, there is little to be gained by such a strategy. The intermediate and late integration strategies using a NMC are better options.

### Expression and Clinical Features Perform Equally Well

A major selling point of existing gene expression based classifiers is their superior performance compared to the existing clinical models. However, we observe a small performance advantage for the NMC trained on C compared with the NMC trained on E (Figure 2. This difference isn't significant, see Figure 3 indicated with a ‘D’. We claim that this might be explained by the more extensive set of clinical parameters that we used. To test this, we split the clinical features into three groups (see Table 1): Original (O, those available at the time the first signatures were published, e.g. grade, age, ER status, etc.), Signatures (S, outputs of clinical models, e.g. NPI, StGallen, etc.), and New (N, those not published before, e.g. matrix formation, central fibrosis, etc.). We repeated the classification experiments, using an NMC with these different sets of clinical variables. We already tested an NMC using the O+S+N features (‘NMC; None C’), and added NMCs using the O+S features, O+N features and only the O features. The result is shown in Figure 6A. Indeed, the NMC classifier using only the original features (O), performs significantly worse than all other options (Figure 6B). Adding the outputs from the clinical models or new features improves the performance (variants using O+S or O+N), and using all three (O+S+N) gives another large improvement. Thus, by including the outputs from clinical models and the new set of clinical features, the performance of the NMC trained on all clinical features is equivalent to that of the NMC trained on E. Therefore, there is no significant performance argument to choose one over the other.

**Figure 9:**
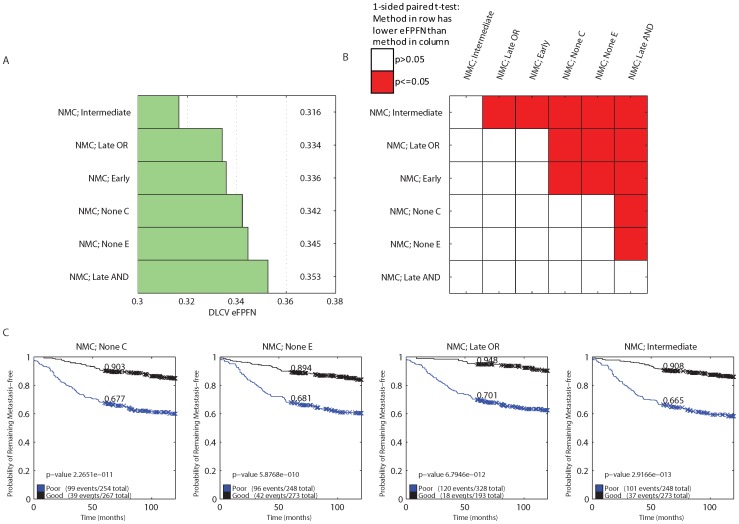
Error rate and KM curves for the NMC classifier with all integration strategies applied to four independent dataset. A) Bar plot indicating the average DLCV eFPFN errors obtained using the NMC classifier with different integration strategies, and types of input data. These results were obtained using the 521 cases from the four independent datasets. B) Overview of all pairwise comparisons of the NMC classifiers, by means of a one sided, paired t-test, testing the hypothesis that the error associated with the approach listed in the row is lower than the error associated with the approach listed in the column. Red cell shading indicates a p-value smaller than 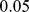, and white cell shading indicates that the p-value was larger than 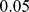. C) Kaplan-Meier curves of the NMC classifier without integration, and the intermediate and late OR integration strategy. We’ve indicated the p-value from the logrank test, and the fraction at five years.

**Figure 10:**
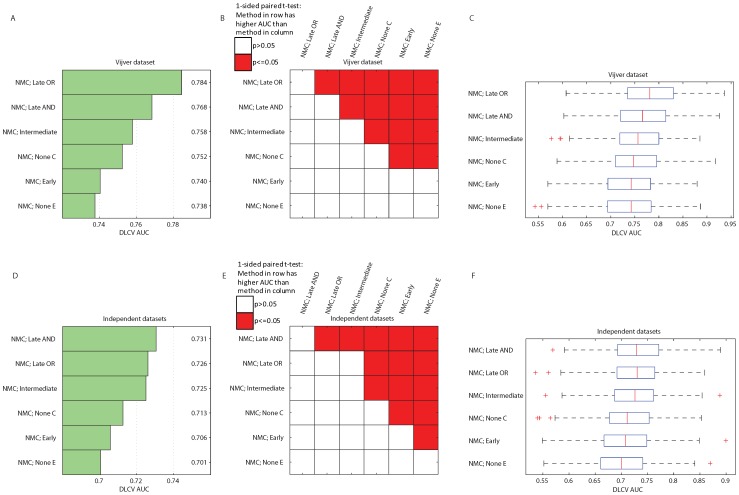
DLCV AUC performances on the Vijver dataset (A, B, C), and independent validation datasets (D, E, F). A, and D) Bar plot indicating the average DLCV AUC obtained using the NMC classifier with different integration strategies, and types of input data. B, and E) Overview of all pairwise comparisons of the NMC classifiers, by means of a one sided, paired t-test, testing the hypothesis that the AUC associated with the approach listed in the row is higher than the AUC associated with the approach listed in the column. Red cell shading indicates a p-value smaller than 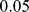, and white cell shading indicates that the p-value was larger than 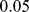. C, and F) Boxplots of the individual DLCV AUC performances obtained (300 AUCs, from 60 repeats of 5 folds), the red line indicates the median.

### The Selected Features

The NMC with late OR strategy performs the best, and therefore we trained a final classifier on all samples of the Vijver dataset. The number of features was chosen by averaging the number of features that was found to be optimal in the inner loops, resulting in 87 and 9, for the expression and clinical features, respectively (see Figure 7 for a pairwise correlation of all features).

First of all, we performed an enrichment analysis for the 87-gene signature. We collected gene sets from GO, KEGG, Reactome, WikiPathways, and the Molecular Signature Database C2 (MSigDB), giving a total of 4525 gene sets with at least five genes. We used the hypergeometric test to asses the significance of the overlap, followed by a Bonferroni correction. A heatmap of the enrichment is shown in Figure 8. The most highly enriched gene set is the van’t Veer signature [1] from MSigDB. This is to be expected, since there is sample overlap between the datasets from Veer *et al.* [1] and Vijver *et al.* [10] (nevertheless it is a positive control). Other than that, many proliferation associated gene sets are enriched. This has previously been identified as a category picked up by most signatures [17].

The nine clinical variables that were selected are shown in Table 2. The set of clinical variables includes a proliferation signature (Mitos, Grade02, Grade07). Moreover, it contains some of the hormone associated variables known to be associated with survival (ER, ERbin, PR). In addition, the outputs from some of the clinical models were selected (NPI Score, Clin NPI). Matrix has not been previously associated with survival.

In order to see whether the clinical features pick up a signal different from the expression features, we inspected their correlation. Figure 7 shows three Subgroups ‘A’, ‘B’ and ‘C’ of correlated genes. Subgroups A and B are correlated with the grade/signature clinical variables (Mitos, grade02, grade07, NPIscore, ClinNPI). In addition, the smaller set of genes in Subgroup C, are correlated with the ER and PR clinical variables (ER, ERbin, PR). This Subgroup is anti-correlated with Subgroups A and B. We performed the same enrichment analysis on these three subgroups of genes, see Figure 7. The genes in Subgroup A are clearly highly enriched for proliferation associated gene sets, which also confirms the positive correlation with proliferation associated clinical parameters.

The SCUBE2 gene from the smaller Subgroup C is part of the ‘Estrogen genes’ in the signature from Paik *et al.* [36]. This explains the positive correlation with the ER and PR clinical parameters. However, the genes in Subgroup C are not enriched for any gene sets (see Figure 7). ER regulates many genes, resulting in very large ER associated gene sets. As a consequence, the set of genes in Subgroup C is probably too small to be able to become significantly enriched.

The matrix variable is not correlated with any of the 87 genes, nor with the other eight clinical variables. Next, we tested whether any of the genes is associated with the matrix clinical variable, by means of a t-test. After Bonferroni correction, none of the genes have a significant p-value (at 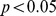). Thus, the information of the matrix variable is not captured by the expression data at all.

### Integration Also Improves Performance on Four Independent Breast Cancer Datasets

The amount of clinical data that is published for breast cancer microarray datasets is often limited. Therefore, a direct validation of the ‘NMC; Late OR’ classifier on independent data is impossible (due to missing clinical features). However, from a previously gathered collection of breast cancer datasets [17], we extracted a total of 521 cases for which survival and seven clinical variables were present (see Materials and Methods section). The NMC classifier with all integration strategies was applied on this dataset, employing the DLCV procedure with the same settings as used for the Vijver dataset (see Materials and Methods section). The other classifiers were omitted since the NMC classifier performed best on the Vijver dataset.

Figure 9a shows the DLCV error rates, and Figure 9b shows their pairwise comparison, revealing that the ‘NMC; Intermediate’ strategy performs the best, followed by the ‘NMC; Late OR’ and ‘NMC; Early’ strategies. Thus, the integration strategies also improve the performance on these four independent datasets. Moreover, the NMC classifiers trained using the expression or clinical data alone perform equally well (eFPFN of 0.342 vs 0.345, no significant difference). Figure 9c shows the Kaplan-Meier curves of these classifiers trained using expression or clinical data alone, showing very similar curves. In addition, employing the ‘NMC; Late OR’ strategy primarily provides a purer good group (0.948 vs 0.903 and 0.894 respectively). The superior performance of the integration strategies, and the equivalent performance of the expression and clinical features confirm our findings on the Vijver dataset.

### Integration Results in Higher AUC Performance

In the DLCV procedure, we optimized the number of features by minimizing the eFPFN error. As an alternative, we repeated the experiments aiming to maximize the AUC, which reflects the performance across the entire ROC curve rather than a single operating point (see Materials and Methods). We repeated the experiments using the NMC classifier, as that classifier achieved the best performance in the eFPFN experiments. All DLCV settings were kept the same (60 repeats, 5 folds, etc.). Figure 10 shows the average AUC results, a pairwise comparison of the classifiers, and boxplots of the AUC results. On both the Vijver dataset, and the independent validation datasets, a late integration strategy achieves the highest AUC. Thus, we conclude that integration also improves the AUC performance.

## Discussion

For all classifiers tested, we found evidence to support the hypothesis that integration of expression and clinical data leads to better predictors. We hypothesize that this is the result of two effects. First of all, both individual classifiers pick up a noisy proliferation associated signal, and their redundancy leads to a better prediction. Secondly, the clinical set of features has some additional information, for example the ‘Matrix formation’ variable, which is not captured by the expression. This complementarity of features results in a synergetic effect on the classification performance.

The late OR integration is the strategy that most often leads to the best performance improvement on the Vijver dataset. Using the late OR strategy, samples for which the individual classifiers are discordant are assigned to the poor outcome group. As a result, the identified good group becomes smaller but also purer. We hypothesize that this is also why the performance increases, the two data types are primarily synergetic in finding a pure group of good cases. A similar effect was seen when combining the classifier outputs of existing gene expression signatures [17]. The intermediate and late OR integration strategy perform the best on the four independent datasets. On these datasets, the late OR strategy also results in a clear improvement in the ten year survival of the good group. Identifying a very pure good outcome group may clinically be the most interesting, since those patients could be spared treatment.

Using the eFPFN as criterion shows that the Late OR strategy is the best on the Vijver data, and the intermediate strategy on the independent data (the Late OR is second best). When using the AUC as criterion, the Late OR strategy performs the best on the Vijver data, and the late AND strategy on the independent data (the Late OR is second best). These differences in the best integration strategy may be due to 1) potential differences in composition of the samples between the cohorts, 2) the use of different microarray platforms, 3) differences in clinical data that is available (much more extensive for the Vijver dataset), and 4) differences in annotation (such as differences in grading between pathologists). Some or all of these effects will play a role in which classifier/integration strategy performs the best. Remarkably, in all cases best performances are achieved by integrating the two data types, showing strong evidence of their synergy.

In the intermediate and late integration strategies, the optimal sets of features are selected on each data type separately and not in the context of the final integrated classifier, which might be sub-optimal. We did not explore alternative feature selection procedures, which take this complementarity into account, due to the additional computational complexity.

The nearest mean classifier significantly outperforms all other classifiers. Thus, our results support earlier indications that a relatively simple classifier, is least hampered by the small sample size problems. On top of that, we conclude that this is the case regardless of the choice of integration strategy. We would like to stress that these claims can only be made for the breast cancer data sets examined in this study.

Gevaert *et al.* [11] also investigated the three types of integration strategies, albeit with only one classifier (Bayesian network). Their conclusion that intermediate and late integration perform better are confirmed in this study. In addition, we show that this is the case without preselecting genes, without discretizing the expression data, and on a larger dataset.

Daemen *et al.* [14] also employed the SVM with intermediate integration, using the same type of kernel (cosine correlation distance). They conclude from their AUC measurements that the SVM trained on clinical data alone performs better than the SVM using intermediate integration, which, in turn, performs better than the SVM trained on the expression data only. Our results show the exact same order in performances. In addition to that, we also conclude that the SVM intermediate and clinical only perform significantly better than the SVM on expression data only. The best option identified in our study, an SVM with late OR integration, was not tested by Daemen *et al.* [14]. However, our analysis convincingly shows that the choice of using an SVM with this type of kernel is rather poor for this type of dataset, since it is outperformed by several other classifiers.

‘Hormone related’ and ‘Proliferation’ features are selected by both the E and C classifiers indicating the importance of these processes in predicting breast cancer outcome. Matrix formation was selected on the Vijver dataset but was not available on other validation datasets. Scoring additional histo-pathological features on tumor specimens may yield further improvement in breast cancer outcome prediction and is therefore worth pursuing.

## Supporting Information

Table S1 **Overview and details of the clinical variables used for the Vijver dataset.**
(XLSX)
